# Different phenotypic plastic responses to predators observed among aphid lineages specialized on different host plants

**DOI:** 10.1038/s41598-019-45220-0

**Published:** 2019-06-21

**Authors:** Arnaud Sentis, Raphaël Bertram, Nathalie Dardenne, Felipe Ramon-Portugal, Ines Louit, Gaël Le Trionnaire, Jean-Christophe Simon, Alexandra Magro, Benoit Pujol, Jean-Louis Hemptinne, Etienne Danchin

**Affiliations:** 1 UMR-5174; EDB (Laboratoire Évolution & Diversité Biologique), CNRS, Université Toulouse III-Paul Sabatier, IRD, 18 route de Narbonne, F-31062, Toulouse, Cedex 9 France; 2 UMR 1349; IGEPP (Institut de Génétique, Environnement et Protection des Plantes); INRA, Agrocampus Ouest, Université Rennes 1; Domaine de la Motte B.P. 35327, F-35653 Le Rheu cedex, Rennes, France; 3 IRSTEA, Aix Marseille Univ., UMR RECOVER, 3275 route Cézanne, 13182 Aix-en-Provence, France; 4 PSL Université Paris, EPHE-UPVD-CNRS, USR 3278 CRIOBE, Université de Perpignan, 52 Avenue Paul Alduy, 66860 Perpignan, Cedex France

**Keywords:** Food webs, Evolutionary ecology, Evolutionary developmental biology

## Abstract

The role of intraspecific variation in the magnitude and direction of plastic responses in ecology and evolution is increasingly recognized. However, the factors underlying intraspecific variation in plastic responses remain largely unexplored, particularly for the hypothesis that the herbivores’ phenotypic response to predators might vary amongst lineages associated with different host plants. Here, we tested whether plant-specialized lineages of the pea aphid, *Acyrthosiphon pisum*, differed in their transgenerational phenotypic response to ladybird predators (i.e., the asexual production of winged offspring by wingless mothers). In a full factorial laboratory experiment, we found that six aphid clonal lineages each specialized either on alfalfa or clover significantly differed in their transgenerational phenotypic response to predators. Some lineages produced an increased number of winged aphids in predator presence while others did not respond. Aphid lineages specialized on alfalfa had stronger phenotypic responses to predators than those specialized on clover. Although we tested only six aphid lineages from two biotypes, our results imply that intraspecific variation in prey phenotypic response of herbivores to predators differs amongst lineages specialized on different host plants. Our findings therefore raise the question of the influence of plant specialization in shaping herbivore phenotypic responses, and highlight the need to consider multi-trophic interactions to understand the causes and consequences of intraspecific variation in complex phenotypic traits.

## Introduction

One promising avenue to better understand the ecology and evolution of plant-herbivore and herbivore-predator interactions is to go beyond bi-trophic interactions and consider adjacent trophic levels^1,2^. Following this idea, authors proposed a tri-trophic niche conceptual framework for understanding herbivore community structure, population divergence, and evolutionary diversification^1,3^. Singer and Stireman^3^ argued that an explicit tri-trophic view of the community structure and diversification of phytophagous insects may explain general ecological and phylogenetic patterns that are currently only partially explained in a bi-trophic perspective. Along the same line, Price *et al*.^1^ emphasised the importance of considering plant traits, abundance, and spatial distribution to better understand predator-prey interactions and the evolution of herbivore traits. This tri-trophic view has proven valuable for explaining intraspecific variation in herbivore resistance to pathogens and parasitoids^4,5^. For instance, Starks *et al*.^6^ compared the effect of a parasitoid, *Lysiphlebus testaceipes*, on the wheat aphid, *Schizaphis graminum*, population growth rate when raised on resistant or susceptible varieties of barley. They found that aphid population growth rates were lower on the resistant barley which resulted in higher parasitism rates compared to aphids on the susceptible barley variety. Such studies suggest that considering plant traits as well as the evolutionary adaptation of herbivore to plants is important to better understand the effect of natural enemies on herbivores’ populations.

Phenotypic plasticity, the capacity of a genotype to express variable phenotypes in different environments^7^, is a common response to environmental variation that can modulate the physiology, morphology, and behaviour of individuals^8–10^. These phenotypic responses influence species interactions^8,11^, as well as the speed and direction of trait evolution^9,12,13^. Plastic responses to predators are common in many taxa and play an important role for predator and prey population dynamics and coevolution^14^. These responses, often referred to as trait-mediated or non-consumptive effects^15,16^, can encompass behavioural changes such as habitat shift, reduction in activity, altered feeding rate as well as changes in life history traits and morphology (e.g., defensive structure, colour, shape)^14,17–23^. Phenotypic responses to predation can be transgenerational, whereby offspring have an altered trait or a distinct alternate phenotype in function of the parental environment^19^. Such transgenerational phenotypic responses are well described in the water flea *Daphnia pulex* and the pea aphid *Acyrthosiphon pisum* where exposure to predator or parasite cues can produce offspring developing defensive crests and spines in the former, and winged dispersing forms in the latter^19,21,24,25^. These facultative morphological changes may be adaptive as they reduce the probability of predation^26,27^. More generally, non-consumptive effects can have strong impacts on the dynamics of interacting species and communities by influencing the behaviour and phenotype of the prey and their offspring^28,29^. Previous studies reported that the magnitude and direction of transgenerational phenotypic responses to predators vary among individuals and/or populations of the same species^18,19,30^. However, plastic responses to predators have been mainly studied from a bi-trophic perspective and not from a tri-trophic perspective. As a result, whether plants and herbivore’s evolutionary adaptation to plants might play by a potential role in herbivore’s plastic responses to predators remains largely undocumented.

Here, we experimentally investigated whether pea aphid clonal lineages specialized on different host plants (i.e. host races or biotypes) differed in their transgenerational phenotypic response to ladybird predators (i.e., the asexual production of winged offspring by wingless mothers). Using a plant–aphid–ladybeetle system, we conducted a full factorial laboratory experiment where six genetically distinct aphid clonal lineages (i.e. asexually reproducing aphid genetic lines) specialized either on alfalfa or clover (referred hereafter to as *Alfalfa* and *Clover* biotypes) were reared on the universal host plant *Vicia fabae* for 10 days in either the presence or absence of ladybird predators. After this period, we removed predators and monitored population density and proportion of winged individuals in each aphid lineage (three lineages per biotype). A standard set of seven microsatellite loci was used to confirm that each lineage represented a unique genotype (clone) and that each belonged to the aphid biotype corresponding to the plant from which it was collected^31^ before being brought back to the laboratory. Our study sought to elucidate whether (1) genetically distinct aphid lineages differed in their transgenerational plastic response to predators, (2) biotypes characterised by different histories of host-plant specialization differed in their plastic responses and (3) aphid plastic response to predators depends on their vulnerability to predators. Following the tri-trophic framework proposed by Price *et al*.^1^ and McPeek^32^, plants that promote predation either by attracting predators or enhancing their predation efficiency should stimulate the evolution of herbivore defences against predators. Predator attraction and predation pressure on aphids are higher for alfalfa than for clover crops^33^. Even in the absence of extensive lineage replication within biotypes allowing us to conclude and generalise on the evolutionary significance of this mechanism, we thus expected aphids from the *Alfalfa* biotype to show stronger plastic responses to predators than aphids from the *Clover* biotype.

## Results

**Figure 1:**
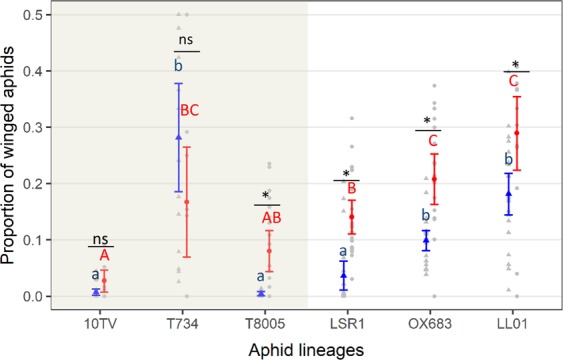
Winged aphid proportion (mean ± 95% CI) with predators (red dots) and without predators (blue triangles) for each aphid lineage (*n* = 20 replicates per treatment). Shaded area: aphid lineages of the *Clover* biotype; Non-shaded area: lineages of the *Alfalfa* biotype. Small or capital letters denote significant differences (*P* < 0.05) among lineages without or with predators, respectively. Asterisk or “ns” denotes significant (*P* < 0.05) or non-significant (*P* > 0.05) predator effect for each lineage (significance levels estimated with post hoc Tukey tests).

We first analysed whether winged aphid proportions differed among aphid lineages and predator treatments. We found that the proportion of winged aphids was affected by the interaction between aphid lineage and predator treatment (*χ*^2^ = 75.17; df = 5; p < 0.0001). Predators significantly increased the proportion of winged aphids although this effect differed among lineages in that it was non-significant for *Clover* lineages T734 and 10TV but significant for the other lineages (Fig. 1). Moreover, in both the presence and absence of predators, the proportion of winged aphids differed among aphid lineages (Fig. 1). We calculated the broad-sense heritability of the proportion of winged offspring produced by wingless adults in control and predation treatments and found that the production of winged offspring was highly heritable with heritability values (±95% CI) of 0.69 ± 0.12 without predators and 0.59 ± 0.30 with predators.

**Figure 2:**
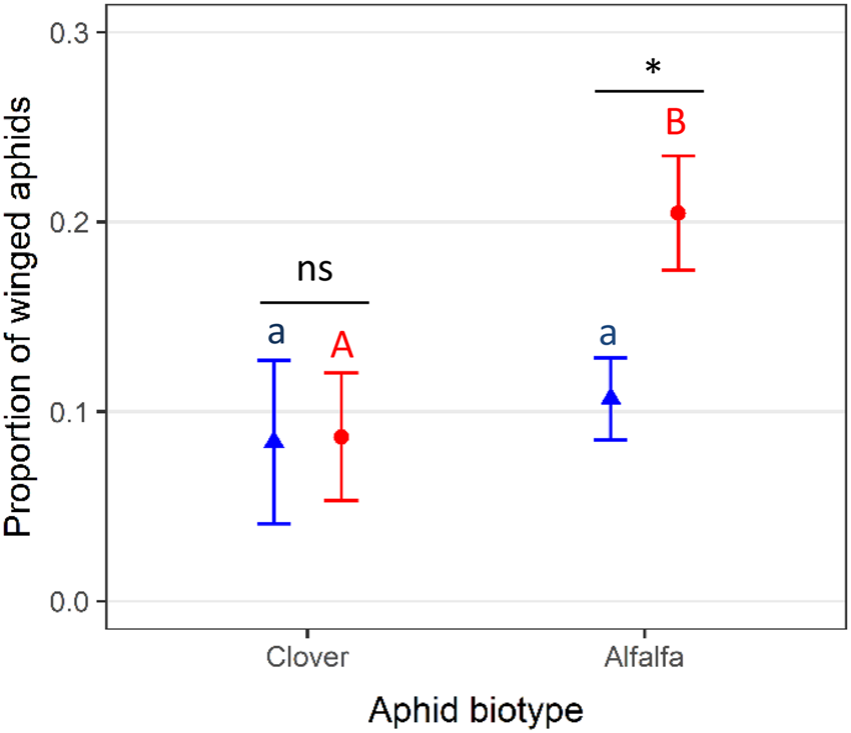
Winged aphid proportion (mean ± 95% CI) with (red dots) or without (blue triangles) predators for the *Clover* and *Alfalfa* biotypes (*n* = 60 replicates per treatment). Small or capital letters denote significant differences (*P* < 0.05) between biotypes without or with predators, respectively. Asterisk or “ns” denotes significant (*P* < 0.05) or non-significant (*P* > 0.05) predator effect for each aphid biotype (significance levels estimated with post hoc Tukey tests).

In view of these differences in transgenerational phenotypic response to predators (Fig. 1), we next analysed whether the among-lineage variation is linked to aphid biotypes using a GLMM with aphid lineage (random factor) nested within aphid biotype. We found that the interaction between predator and aphid biotype was related to the proportion of winged aphids (*χ*^2^ = 10.07; df = 1; p = 0.0015). The presence of predators leads to a two-fold increase in winged aphid proportion for the *Alfalfa* biotype but this effect was not significant for the *Clover* biotype (Fig. 2).We also reanalysed the data by removing the aphid lineage T734 because in this lineage, the production of winged aphid was slightly lower in presence of predator which could have driven an overall significant interaction. Without T734, the interaction between biotype and predator (χ2 = 27.43, df = 1, p < 0.0001) was still significant, thereby indicating that our results were not driven by a single lineage that would differ from the other five.

**Figure 3:**
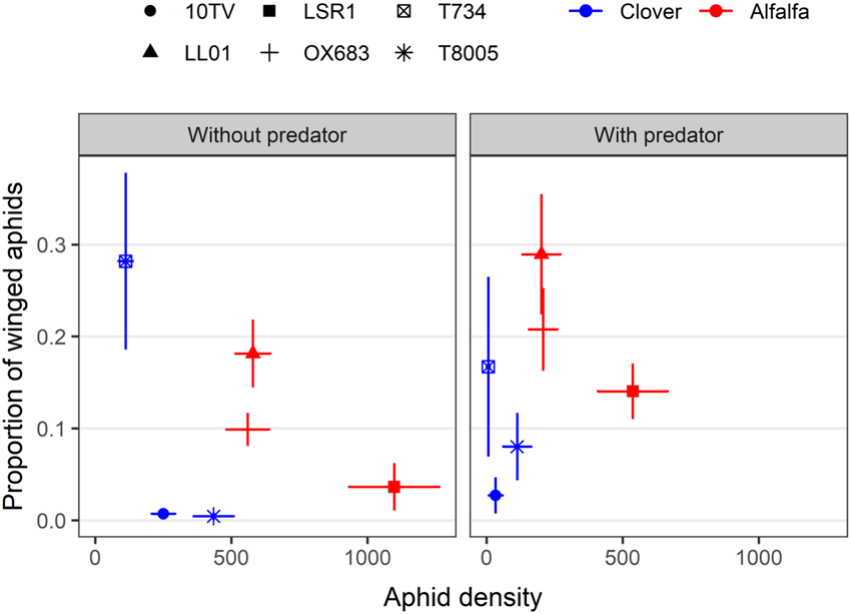
Relationship between aphid density (mean ± 95% CI) and winged aphid proportion (mean ± 95% CI) without (left panel) or with (right panel) predators for each aphid lineage. Each dot represents one aphid lineage. In blue: *Clover* biotype. In red: *Alfalfa* biotype.

To investigate the link between aphid population density and winged aphid proportion, we calculated for each lineage the mean aphid density and winged aphid proportion with and without predators. We next arcsine square root transformed proportions to linearize their binomial distribution^34^ and used an ANCOVA to analyse the relationship between aphid population density and winged aphid proportion with and without predators. We found that the proportion of winged aphids was not related to aphid density (*F* = 0.07; df = 1; p = 0.7910) in both the presence and absence of predators (interaction predation × aphid density: *F* = 0.50; df = 1; p = 0.4985). In other words, in our experimental system, there was no significant increase in the proportion of winged aphids with increasing aphid density across aphid lineages. In particular, some lineages such as LSR1 reached high densities but produced a low proportion of winged aphids (Fig. 3). Lineages of the *Alfalfa* biotype (LSR1, LL01, and OX683) reached higher densities than lineages of the *Clover* biotype (T734, 10TV, and T8005) both with and without predators (Fig. 3). Aphid density was the lowest for the lineage T734 and the highest for the lineage LSR1 in both predation treatments.

**Figure 4:**
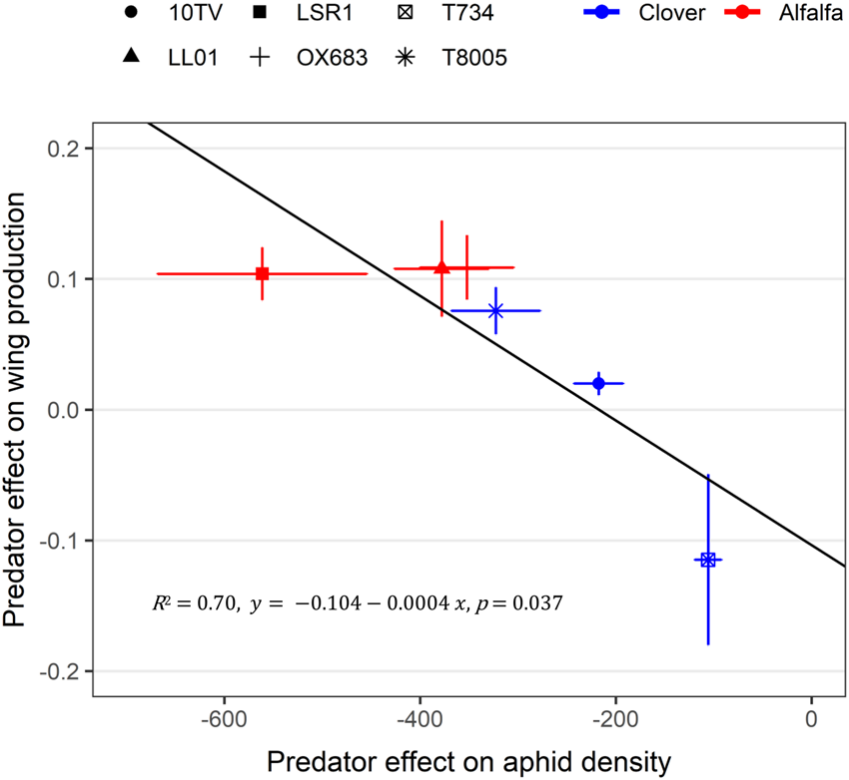
Relationship between the effect (mean ± se) of predators on aphid density (*X* axis) and their effect (mean ± se) on winged aphid proportion (*Y* axis). Each dot represents one aphid lineage. In blue: *Clover* biotype. In red: *Alfalfa* biotype.

We also investigated for each aphid lineage the relationship between the effect of predators on aphid density (i.e. mean density with predators – mean density without predators) and their effect on winged aphid proportion (i.e. mean proportion with predators – mean proportion without predators) using a linear model. We found that the increased proportion of winged aphids in response to predation was proportional to the effect of predator on aphid population size: the stronger the aphid density reduction due to predation, the stronger the increase in winged aphid proportion (Fig. 4). Finally, the reduction of aphid population size due to predation was significantly linked to aphid population size in the absence of predators: the higher the aphid density in the absence of predators, the stronger the aphid density reduction (Fig. S1).

## Discussion

While herbivore evolutionary divergence linked to ecological specialisation has received considerable attention^1,31^, its importance for prey phenotypic response to predators remains largely unexplored. Here, we found that aphid lineages differed in their transgenerational phenotypic response to predators. We then investigated whether host plant specialisation could potentially contribute to explain variation amongst lineages. Our results based on three lineages per biotypes were in agreement with the hypothesis that aphids specialized on alfalfa had a stronger phenotypic response to predators than those specialized on clover. Although this is no material for generalisation, our finding that variation between biotypes exists is proof of concept evidence. It is an important first step for our understanding of herbivore’s evolutionary adaptation to plants and its potential role in herbivore’s plastic responses to predators. Our finding calls for testing the general evolutionary significance of such hypothesis. It also implies that, as outlined in previous studies^1,3,32^, considering tri-trophic interactions, and thereby a third partner such as the host plant, could bring a better understanding of herbivore trait variation and evolution.

Previous studies reported examples of variation in herbivore phenotypic responses to predators^18^, including inter- and intraspecific variation in the production of winged aphid offspring^21,35–37^. Accordingly, we found that aphid clonal lineages differed in sensitivity to predators: some lineages produced a high proportion of winged aphids in response to predators, whereas others showed no differences in presence or absence of predators. Few studies have investigated the causes of intraspecific variation in wing production among aphid lineages in response to predators. Crowding is well known to induce winged aphids^36,38,39^ and could explain variations among lineages when they have different population growth rates and thus crowding levels (i.e. fast developing populations crowd faster than slow developing ones)^37,40^. In our experiment, the initially introduced females producing many offspring could have thus perceived a potential crowding stress earlier than those producing less offspring. However, we found no effect of aphid density on winged aphid proportion in our study indicating that the observed difference among clones in the production of winged aphids was not related to a crowding effect. Increased movements and physical contacts with conspecifics are the proximal cause for the producing winged offspring in the pea aphid^41^. It could be that the clones tested in this study differ in behaviour. Previous work showed strong variation in behavioural responses to aphid alarm pheromone between *A. pisum* clones specialized on *pea* (*Pisum sativum*) vs *Alfalfa* and *pea* vs *Clover*, but not between *Alfalfa* and *Clover* biotypes^42^, leaving unresolved our observed differences.

Our results indicate that variation in response to predators among aphid lineages varied with the aphid biotype. The proportion of winged aphids significantly increased in response to predators in the *Alfalfa* biotype but not in the *Clover* biotype. Previous studies reported that aphid biotypes vary in their secondary symbiont community^43–45^, resistance to pathogen and parasitoids^4,5^, defensive behaviour and susceptibility to predators^33^. Although we used only three aphid lineages per biotype, our study builds upon previous findings by showing that the aphid biotypes contribute to explain some variation in plastic response to predators. As aphid biotypes are genetically differentiated^31,46,47^ in a way that affects their performance on different host plants^46–48^, it is likely that host-plant linked adaptive divergence has the potential to shape prey phenotypic response to predators. Our results do not bring evidence for a general evolutionary significance of this mechanism. However, they bring proof of concept evidence for its potential by showing that the link between the plastic response and the biotype can exist. Moreover, we estimated relatively high intercolonial heritabilities for the production of winged offspring, indicating a potential for evolution by selection, which also supports this potential.

Although within-biotype variation should not be omitted, our results raise the question of why the two pea aphid biotypes show contrasting responses to predators. Theory predicts that inducible defences evolve only if benefits outweigh costs implying that, in the presence of predators, the fitness of plastic individuals must be higher^14,20,29^. For plasticity to be adaptive the benefits of producing wings should thus outweigh the costs of developing wings. According to the tri-trophic view^1^, plants attracting few predators or on which predators are inefficient at finding or catching prey should not lead to the evolution of strong plastic responses by herbivores. One hypothesis is that differences in phenotypic plasticity among biotypes may result from trade-offs in defensive response that vary according to the host plant. Predation pressure on aphids are acknowledged to vary among host plants^49^. It is therefore only logical that host plants with low predation pressure host herbivores with weak plastic responses to predators. A previous study reported that predation pressure on aphids is weaker on *Clover* than on *Alfalfa* plant^33^, which supports this hypothesis. We used three aphid lineages per biotype, which implies that we did not directly test for the effects of plant-herbivore interactions on the evolution of wing plasticity in aphids. Our comparison of two groups (of three) aphid genotypes that belong to different host-plant specialized populations, and were reared experimentally on a universal host plant, likely reflects genetically-based differences in predator response between biotypes. Our finding thus supports a potential role of selection driven by plant-herbivore-enemy interactions. However, these differences may also result from other selective pressures or from drift. Our study is thus a first exploratory step towards the determination of the role of host plant specialisation for phenotypic response to predators. Extending our approach to a wider diversity of aphid lineages and biotypes collected along a gradient of predation pressure is needed to confirm and assess the general significance of such findings.

A convergent hypothesis is that variation of the phenotypic response to predators among aphid biotypes is driven by their relative performance on the host plant and thus their adaptation to the host plant. This hypothesis is supported by our experimental data showing that (1) aphid lineages of the *Alfalfa* biotype reach higher densities (i.e. perform better) than those of the *Clover* biotype on the universal host, (2) the impact of the predators on aphid density is proportional to aphid population performance with larger aphid populations suffering stronger reduction than smaller ones and (3) the production of winged offspring in response to predators is positively correlated with the reduction of aphid populations by predators. Altogether, our work shows that lineages of the *Alfalfa* biotype are more impacted by predators than those of the *Clover* biotype, which, in turn, leads to a stronger increase in winged offspring production in response to predators. This indicates that the relative performance of the biotypes statistically explains the variation in their transgenerational phenotypic response to predators. The performance of aphid biotypes on different host plants is genetically determined and results from evolutionary adaptation to the host plant^46–48^. Although the cost-benefit trade-offs involved in herbivore co-adaptation to host plants and predators remain to be investigated in more detail, our results suggest that selection on aphid performance by the plants is, at least partly, linked to the selection exerted on aphid phenotypic plasticity by predators. Therefore, adaptation to the host plant may promote divergent phenotypic responses to predators in pea aphids.

## Conclusion

Phenotypic plastic responses of prey to their predators are important examples of trait-mediated or sublethal predator effects that significantly influence direct and indirect interactions which, in turn, can impact food web structure and dynamics^29,50–53^. A key issue to better understand the role of trait-mediated effects for eco-evolutionary dynamics of predator and prey populations is thus to determine the factors explaining intraspecific variation in prey phenotypic responses to predators. Here, we showed that prey lineages vary in their phenotypic responses to predators. Our findings, by showing that plasticity varies between biotypes represented by a limited number of lineages, also open the question of a potential role of host plant specialisation in this type of variation. Although the proximate mechanisms responsible for such differences among biotypes remain to be investigated in more detail, differential adaptation to host plants is a potential explanation. Our findings question the role of herbivore evolutionary adaptive history to the host plant in shaping herbivore phenotypic responses to predators and highlight the importance of considering multi-trophic interactions to better understand the causes and consequences of intraspecific variation in complex phenotypic traits.

## Materials and Methods

### Biological model

The pea aphid *Acyrthosiphon pisum* Harris (Hemiptera: Aphididae) has been commonly used as a model system for the study of ecological speciation^31,54,55^ and phenotypic plasticity^56^. Pea aphids feed on many Fabaceae species and form genetically differentiated populations (“biotypes”) that are specialized on different host plants^31,46,47^. Host-plant specialization (i.e. higher affinity to feed and reproduce on a particular plant species) reduces gene flow among host-plant associated populations and affects their relative performance (i.e. fecundity and population growth rate) depending on which host plant they are feeding on^46–48^. Nevertheless, all biotypes can feed and successfully develop on a universal legume host, which is the broad bean, *Vicia faba*^31^.

The pea aphid is attacked by several fungal, parasitoid, and predator species and responds to these natural enemies behaviourally, physiologically and morphologically^30,57,58^ with the possible involvement of facultative symbionts^59^. The most spectacular response is probably the asexual production of winged offspring that disperse and thus escape predators^26,30^. Other environmental factors such as crowding, low plant quality, and temperature can also induce the production of winged offspring^21,30,58,60,61^. Altogether, previous studies suggest that wing induction is a general adaptive response to stress in aphids which increases the prevalence and persistence of aphid clonal populations^26^.

### Experimental system

The experimental system is a three level food chain: the predatory ladybird *Harmonia axyridis*, the pea aphid *A. pisum*, and the broad bean *V. faba*. Approximately 200 adults of *H. axyridis w*ere collected in October 2015 in Auzeville-Tolosane (43°32’N, 1°29’E, South of France), brought to the laboratory, reared in 5000-cm^3^ plastic boxes, and fed three times a week with an excess of pollen and pea aphids (Louse_31 lineage). Corrugated filter paper was added to each box to provide a suitable substrate for oviposition. *H. axyridis* eggs were collected three times a week and neonate larvae were reared in 175-cm^3^ plastic boxes and fed pea aphids *ad libitum* before experiments. Stock colonies of 6 pea aphid clonal lineages (Table S1) were maintained for more than three months before the beginning of the experiments at our laboratory at low density on broad bean grown from seeds (Ets Henrion s.a.; Belgium, cv. Aquadulce) in nylon cages (30 × 30 × 30 cm). All aphid lineages were free of any of the eight secondary symbionts reported in the pea aphid^62^ (i.e. only harbour the obligate endosymbiont *Buchnera aphidicola*) to avoid potential confounding effects of variation in symbiont composition among aphid lineages. These lineages were selected from a large collection of clones maintained at INRA Rennes and their symbiotic status was checked using diagnostic PCR as described in Peccoud *et al*.^63^. Three lineages belonged to the *Clover* biotype and three others to the *Alfalfa* biotype and, for each biotype, one of the tested lineages was of green colour whereas the two other lineages were pink (Table S1). We used a standard set of seven microsatellite loci to confirm that each lineage represented a unique genotype (clone) and that each belonged to the aphid biotype corresponding to the plant from which it was collected^31^. All insects and plants were maintained in air-conditioned chambers (Dagard®) at 21 ± 1 °C, 50–60% relative humidity, and under a 16 L:8D photoperiod to mimic spring conditions during which the pea aphid only reproduces by apomictic parthenogenesis (i.e., offspring are clones of their mother).

### Experimental design

In a full factorial experiment, we measured the effects of predators on wing induction in the 6 aphid clonal lineages. Three 8-day-old bean plants with two unfurled leaves were placed in 500 mL plastic pots containing 400 mL of fertilized soil substrate (®Jiffy substrates NFU 44–551), and then enclosed in transparent plastic cylinders (ø: 14 cm; h: 29 cm). The top of the cylinder and the two lateral openings were covered with mesh muslin for ventilation. During the experiment, bean plants were watered every three days with 0.75 mL of tap water per pot. Before the experiment, we maintained each aphid lineage at low density (i.e. 4 adults per plant) for two generations to avoid potential maternal and grand-maternal effects linked to crowding. At the onset of the experiment, we transferred, for each lineage separately, six two-day-old adult clonal female *A. pisum* (obtained from synchronous cohorts) to the upper leaves of the plants using a fine paintbrush, and allowed to acclimatize and reproduce for 24 h. For each of the 6 aphid lineages, we performed 20 replicates without predators and 20 replicates with predators for which one second instar *H. axyridis* larva was introduced into each experimental unit (i.e. plastic cylinder containing one plant). After 10 days (this experimental duration was chosen, based on preliminary experiment, to minimize resource competition linked to plant depletion and to allow offspring of the first generation *F*_1_ to develop as much as possible while preventing their reproduction as they became sexually mature after 10 days in our experimental conditions), we removed the predators and collected all aphids using a fine paintbrush and counted them under a stereoscopic microscope. We recorded the numbers of winged and wingless adults, as well as the numbers of pre-winged and unwinged nymphs. While only adults have fully developed wings, 3^rd^ and 4^th^ instar pre-winged nymphs display wing buds that helped differentiate them from pre-wingless nymphs. As it was logistically not possible to perform all the 240 (6 lineages × 2 predator treatments × 20 replicates) replicates simultaneously, we conducted the experiment over three different dates, each incorporating one third of each treatment. During the experiments, temperature and humidity were recorded continuously using Hobo U12 (Hobo®) units.

### Statistical analyses

We performed the statistical analyses in two steps to (1) investigate whether winged aphid proportion differed among aphid lineages and predator treatments, and (2) determine whether the observed variations were linked to aphid evolutionary divergence (i.e. biotype). We thus first analysed the effects of the presence of predators, aphid lineage, and their interactions on the proportion of winged aphids with a binomial GLMM (Generalized Linear Mixed Model) with experimental dates as a random effect. When testing the model assumptions, model overdispersion was detected and corrected by including experimental units (i.e., plastic cylinders) as a random effect^64^. The significance of the fixed model terms was assessed using Chi-squared tests from analyses of deviance based on maximum likelihood estimates. Post-hoc Tukey tests were used to determine significant differences among means. Second, we investigated the effects of aphid biotype, predator presence and their interaction on winged aphid proportions using a binomial GLMM model as described above, but adding lineage nested in aphid biotype and in capture country and lineage colour as random effects. We decided not to include lineage colour as a fixed factor as the focus of this study was on aphid biotype and also because we had only one green lineage for each aphid biotype which prevented us from testing the interaction between aphid colour and biotype.

To investigate the link between aphid population density and winged aphid proportion, we calculated for each lineage the mean aphid density and winged aphid proportion with and without predators. We next arcsine square root transformed proportions to linearize their binomial distribution^34^ and used an ANCOVA to analyse the relationship between aphid population density and winged aphid proportion with and without predators. We also investigated for each aphid lineage the relationship between the effect of predators on aphid density (i.e. mean density with predators – mean density without predators) and their effect on wing aphid proportion (i.e. mean proportion with predators – mean proportion without predators) using a linear model.

We finally calculated the broad-sense heritability of the proportion of winged offspring produced by wingless adults in control and predation treatments. Phenotypic variance, V_P_ may be partitioned into its environmental V_E_ and genetic components, V_G_, such that V_P_ = V_E_ + V_G_ + Cov_GE_. The genotype-environment covariance Cov_GE_ is considered to be zero in a randomised environment (Falconer, 1989). In clonal organisms, V_G_ can be estimated from the among-lineage variance component (in such case, V_G_ does not only include additive, dominant and epistatic genetic components of variance but also non-genetic components of variance estimating the transgenerational effects of non-genetic inheritance mechanisms), and V_E_ from the within-lineage variance component. For each predator treatment, we estimated variance components (V_G_ and V_E_) using a binomial GLMM including aphid lineage ID as a random effect^65^. We used the variance among clones and the residual variance as estimates of V_G_ and V_E_, respectively. We next calculated the broad-sense heritability as H² = V_G_/V_P_. GLMM were computed using the lme4 package^66^ in R 3.4.1^67^.

## Supplementary information

Supplementary information

## Data Availability

We confirm that the Data supporting the results will be archived in an appropriate public repository such as Dryad or Figshare.
