# Supplementary material for: Different phenotypic plastic responses to predators observed among aphid lineages specialized on different host plants
Source: Sci Rep. 2019 Jun 21;9:9017. doi: 10.1038/s41598-019-45220-0 (PMC6588606; doi:10.1038/s41598-019-45220-0)
Supplement: Supplementary file 1 — Supplementary information [file 41598_2019_45220_MOESM1_ESM.docx]

**Supplementary materials**

**Different phenotypic plastic responses to predators observed among aphid lineages specialized on different host plants.**

Arnaud Sentis^1,3*^, Raphaël Bertram^1^, Nathalie Dardenne^1^, Felipe Ramon-Portugal^1^, Ines Louit^1^, Gaël Le Trionnaire^2^, Jean-Christophe Simon^2^, Alexandra Magro^1^, Benoit Pujol^1,4^, Jean-Louis Hemptinne^1‡^, and Etienne Danchin^1‡^

^1^ UMR-5174; EDB (Laboratoire Évolution & Diversité Biologique); CNRS, Université Toulouse III-Paul Sabatier, IRD, 18 route de Narbonne, F-31062 Toulouse Cedex 9, France.

^2^ UMR 1349; IGEPP (Institut de Génétique, Environnement et Protection des Plantes); INRA, Agrocampus Ouest, Université Rennes 1; Domaine de la Motte B.P. 35327, F-35653 Le Rheu cedex, France.

^3^ IRSTEA, Aix Marseille Univ., UMR RECOVER, 3275 route Cézanne, 13182 Aix-en-Provence, France.

^4^ PSL Université Paris, EPHE-UPVD-CNRS, USR 3278 CRIOBE, Université de Perpignan, 52 Avenue Paul Alduy, 66860 Perpignan Cedex, France.

^‡^ denotes equal authors’ contribution

**Table S1.** Information about aphid lineages’ color, biotype, and capture sites.

| ***Lineage name*** | ***Biotype*** | ***Color*** | ***Collection site*** | ***Capture country*** |
| --- | --- | --- | --- | --- |
| **T8005** | *Clover* | green | Saint-Augustin | Canada |
| **10TV** | *Clover* | pink | Lusignan | France |
| **T734** | *Clover* | pink | Saint-Augustin | Canada |
| **LL01** | *Alfalfa* | green | Lusignan | France |
| **LSR1** | *Alfalfa* | pink | Ithaca | USA |
| **Oxford 683** | *Alfalfa* | pink | Oxford | UK |

**Table S2.** Values of the log odd ratio estimates, standard errors, z statistics, and P values of the GLMMs for the effects of aphid lineage, predator, and their interactions on the proportion of winged aphids. Significant effects are in bold (*P* < 0.05).

| **Fixed effects** | **Estimate** | **s.e** | ***z*** | ***p*** |
| --- | --- | --- | --- | --- |
| Intercept (lineage 10TV without predators) | -5.84 | 0.37 | -15.54 | **< 0.0001** |
| Lineage LL01 | 4.13 | 0.40 | 10.22 | **< 0.0001** |
| Lineage LSR1 | 1.00 | 0.44 | 2.82 | **0.022** |
| Lineage OX683 | 3.55 | 0.43 | 8.19 | **< 0.0001** |
| Lineage T734 | 4.59 | 0.46 | 10.03 | **< 0.0001** |
| Lineage T8005 | -0.38 | 0.49 | -0.77 | 0.441 |
| Predator effect (PE) | 2.30 | 0.57 | 4.04 | **< 0.0001** |
| LL01 × PE | -1.65 | 0.59 | -2.80 | **0.005** |
| LSR1 × PE | 0.59 | 0.66 | 0.90 | 0.367 |
| OX683 × PE | -1.29 | 0.66 | -1.95 | **0.050** |
| T734 × PE | -2.76 | 0.80 | -3.43 | **0.001** |
| T8005 × PE | 1.18 | 0.72 | 1.64 | 0.101 |

Almunia, J., Basterretxea, G., Aristegui, J. & Ulanowicz, R.E. (1999) Benthic-pelagic switching in a coastal subtropical lagoon. Estuarine, Coastal and Shelf Science, 49, 363–384.

Baird, D., Luczkovich, J. & Christian, R.R. (1998) Assessment of spatial and temporal variability in ecosystem attributes of the St Marks National Wildlife Refuge, Apalachee Bay, Florida. Estuarine, Coastal and Shelf Science, 47, 329–349.

Baird, D. & Ulanowicz, R.E. (1989) The seasonal dynamics of the Chesapeake Bay ecosystem. Ecological Monographs, 59, 329–364.

Hagy, J.D. (2002) Eutrophication, Hypoxia and Trophic Transfer Efficiency in Chesapeake Bay. PhD Dissertation. University of Maryland at College Park (USA).

Hall, S.J. & Raffaelli, D. (1991) Food-web patterns: lessons from a species-rich web. Journal of Animal Ecology, 60, 823–841.

Homer, M. & Kemp, W.M. (unpublished) Growth and Development: Ecosystems Phenomenology.

Kitching, R.L. (2004) Food Webs and Container Habitats, the Natural History and Ecology of Phytotelmata. Cambridge University Press, Cambridge.

Krause, A. & Mason, D. A. Krause, PhD. Dissertation. Michigan State University. Ann Arbor, MI. USA.

Lafferty, K.D., Hechinger, R.F., Shaw, J.C., Whitney, K.L. & Kuris, A.M. (2006) Food webs and parasites in a salt marsh ecosystem. Disease ecology: community structure and pathogen dynamics (eds S. Collinge & C. Ray), pp. 119–134. Oxford University Press, Oxford.

Martinez, N.D. (1991) Artifacts or attributes? Effects of resolution on the little rock lake food web. Ecological Monographs, 61, 367–392.

Monaco, M.E. & Ulanowicz, R.E. (1997) Comparative ecosystem trophic structure of three U.S. Mid-Atlantic estuaries. Marine Ecology Progress Series, 161, 239–254.

de Nooy, W., Mrvar, A. & Batagelj, V. (2011) Exploratory Social Network Analysis with Pajek. Cambridge University Press, Cambridge.

Patricio, J. (unpublished) Master’s Thesis. University of Coimbra, Coimbra, Portugal.

Ulanowicz, R.E., Bondavalli, C. & Egnotovich, M.S. (1998) Network analysis of trophic dynamics in South Florida ecosystems, 97: the Florida Bay ecosystem. Annual Report to the United States Geological Service Biological Resources Division, University of Miami Coral Gables, [UM-CES] CBL 98-123. Chesapeake Biological Laboratory, Solomons, MD 20688-0038 USA.

Ulanowicz, R.E., Heymans, J.J. & Egnotovich, M.S. (2000) Network analysis of trophic dynamics in South Florida ecosystems. FY 99: the graminoid ecosystem. Technical Report TS-191-99, Maryland System Center for Environmental Science, Chesapeake Biological Laboratory, Maryland, USA.

Warren, P.H. (1989) Spatial and temporal variation in the structure of a freshwater food web. Oikos, 55, 299–311.

Woodward, G. & Hildrew, A.G. (2001) Invasion of a stream food web by a new top predator. Journal of Animal Ecology, 70, 273–288.

Yodzis, P. (1998) Local trophodynamics and the interaction of marine mammals and fisheries in the Benguela ecosystem. Journal of Animal Ecology, 67, 635–658.

**Table S3.** Values of the log odd ratio estimates, standard errors, z statistics, and P values of the GLMMs for the effects of aphid biotype, predator, and their interactions on the proportion of winged aphids. Significant effects are in bold (*P* < 0.05).

| **Fixed effects** | **Estimate** | **s.e** | ***z*** | ***p*** |
| --- | --- | --- | --- | --- |
| Intercept (*Clover* biotype without predators) | -4.51 | 0.83 | -5.48 | **< 0.0001** |
| *Alfalfa* biotype | 1.85 | 1.13 | 1.64 | 0.102 |
| Predator effect (PE) | 2.10 | 0.32 | 6.41 | **< 0.0001** |
| *Alfalfa* biotype × PE | -1.11 | 0.35 | -3.17 | **0.001** |

**Figure S1.** Relationship between aphid density (mean ± se) without predators (*X* axis) and the effect (mean ± se) of predators on aphid density (*Y* axis). Each dot represents one aphid lineage. In blue: *Clover* biotype. In red: *Alfalfa* biotype.


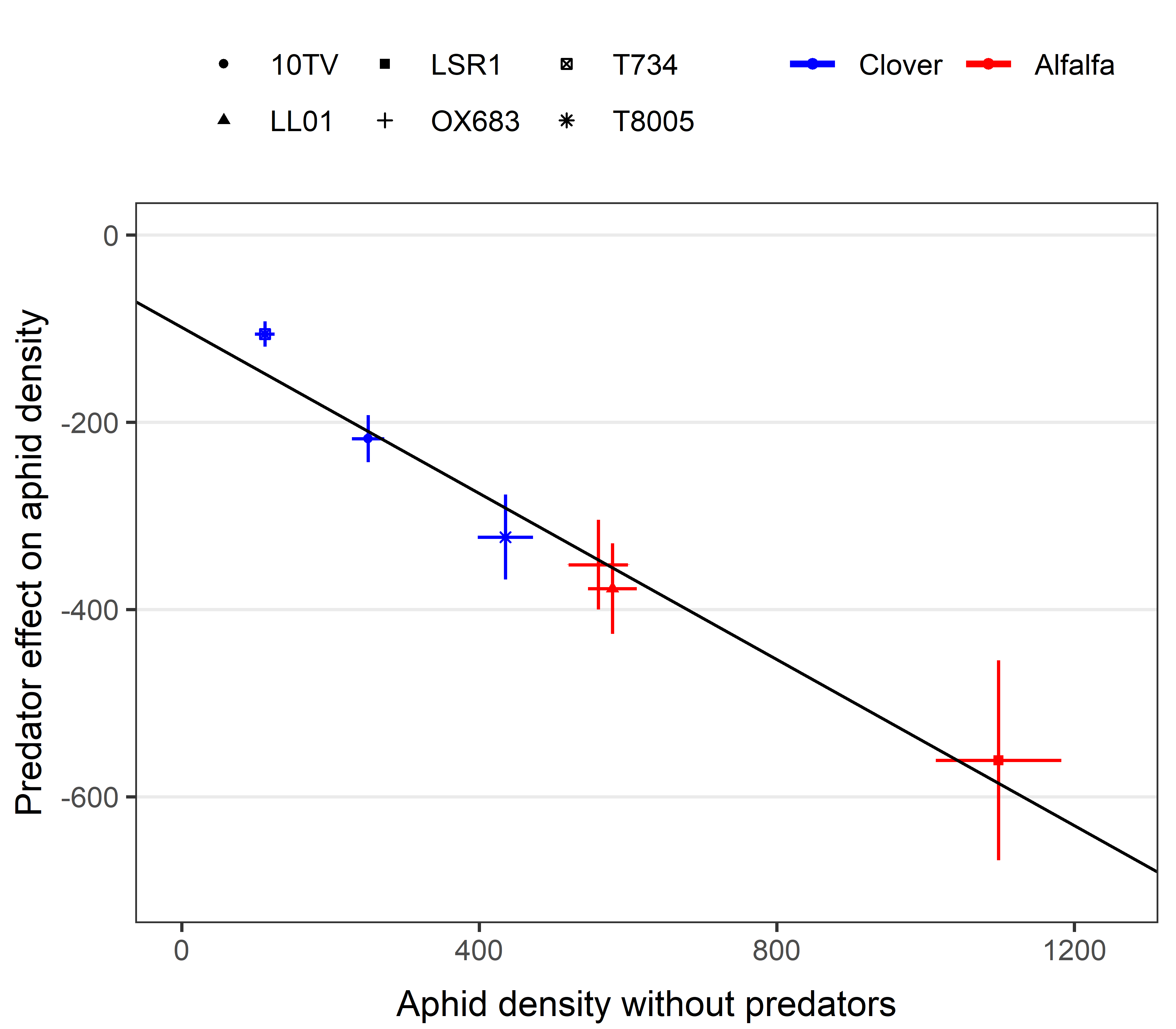


*R*^2^ = 0.96, $y= -98.46-0.44 x$, *p* = 0.0004
